# MicroRNA-146a-loaded magnesium silicate nanospheres promote bone regeneration in an inflammatory microenvironment

**DOI:** 10.1038/s41413-023-00299-0

**Published:** 2024-01-15

**Authors:** Jiakang Yang, Jing Shuai, Lixuen Siow, Jingyi Lu, Miao Sun, Wenyue An, Mengfei Yu, Baixiang Wang, Qianming Chen

**Affiliations:** Stomatology Hospital, School of Stomatology, Zhejiang University School of Medicine, Zhejiang Provincial Clinical Research Center for Oral Diseases, Key Laboratory of Oral Biomedical Research of Zhejiang Province, Cancer Center of Zhejiang University, Hangzhou, 310000 China

**Keywords:** Bone, Bone quality and biomechanics

## Abstract

Reconstruction of irregular oral-maxillofacial bone defects with an inflammatory microenvironment remains a challenge, as chronic local inflammation can largely impair bone healing. Here, we used magnesium silicate nanospheres (MSNs) to load microRNA-146a-5p (miR-146a) to fabricate a nanobiomaterial, MSN+miR-146a, which showed synergistic promoting effects on the osteogenic differentiation of human dental pulp stem cells (hDPSCs). In addition, miR-146a exhibited an anti-inflammatory effect on mouse bone marrow-derived macrophages (BMMs) under lipopolysaccharide (LPS) stimulation by inhibiting the NF-κB pathway via targeting tumor necrosis factor receptor-associated factor 6 (TRAF6), and MSNs could simultaneously promote M2 polarization of BMMs. MiR-146a was also found to inhibit osteoclast formation. Finally, the dual osteogenic-promoting and immunoregulatory effects of MSN+miR-146a were further validated in a stimulated infected mouse mandibular bone defect model via delivery by a photocuring hydrogel. Collectively, the MSN+miR-146a complex revealed good potential in treating inflammatory irregular oral-maxillofacial bone defects.

## Introduction

Oral and maxillofacial bone defects resulting from many causes, including trauma, tumor removal, periodontitis or peri-implantitis, remain a challenge for clinical orthopedic surgery and oral medicine. When injured, bone tissue undergoes three continuing and overlapping regenerative phases: inflammation, regeneration and remodeling.^1^ During the normal bone repair process, inflammation is triggered after injury and quickly resolves to create a proregenerative microenvironment that is enriched with proangiogenic and pro-osteogenic factors to stimulate bone regeneration and tissue repair.^2^ However, unresolved chronic inflammatory conditions were found to negatively impact bone repair and result in increased rates of delayed healing and nonunion of fractures, which were prevalent in patients with osteoarthritis, type I diabetes, obesity, and rheumatoid arthritis.^3^ Due to the distinctive structure of jawbones with teeth, oral and maxillofacial bone defects tend to be irregular and hard to graft with bulky or rigid biomaterials. Complex microbiota colonization in the oral cavity might exist as a source of infection, making the bone defect area chronically infected and impairing the normal healing process.^4,5^ Thus, a practical biomaterial for repairing irregular oral and maxillofacial bone defects with immunoregulatory function via tissue engineering is still in high demand.

MicroRNAs are endogenous small noncoding RNAs that regulate gene expression through post-transcriptional processing.^6^ Studies have shown that miR-146a plays an important role in both immunoregulation^7,8^ and bone homeostasis.^9,10^ By directly targeting TRAF6 and interleukin-1 receptor-associated kinase 1 (IRAK1), two key adapter molecules in the NF-κB pathway, miR-146a negatively regulates the NF-κB-mediated immune response to microbial infection.^11^ With higher expression observed in mouse periodontitis tissues,^8^ miR-146a was reported to directly target Toll-like receptor 4 (TLR4) and downregulate its expression.^12^ On the other hand, miR-146a was demonstrated to be positively associated with the development of ankylosing spondylitis^13^ and to control age-related bone loss in mice.^9^

Nucleic acid drugs require a high-efficiency delivery vector in local medication therapy, such as liposomes, cationic polymers and inorganic particles.^14,15^ By modifying classical monodispersed silica colloidal particles, we fabricated MSNs with a rough surface and hollow mesoporous structure,^16,17^ which accelerated the in vitro osteogenic differentiation of MC3T3-E1 cells with good biocompatibility as well as high cellular uptake efficiency and drug-loading capacity.^17,18^ The osteogenic-promoting effect of MSNs may be attributed to magnesium ions by activating the Wnt/β-actin pathway and upregulating the expression of Runt-related transcription factor 2 (Runx2).^19^ In addition, it was reported that a simple silica-based material (Si(OH)_4_) could increase the expression of endogenous miR-146a in human bone marrow mesenchymal stem cells (hBMSCs) and promote osteogenic differentiation.^20^ Thus, MSNs may be a suitable vector to load miR-146a in promoting inflammatory bone formation with a potential synergetic effect, and the nanosphere structure made MSNs easy to disperse into every corner of an irregular bone defect area.

The objective of this study was to fabricate a novel nanomaterial, MSN+miR-146a, and assess the oligo transfection effect of MSNs as well as the effects of the biomaterial on both osteogenesis and immunoregulation to investigate its potential application value in treating irregular bone defects with an inflammatory microenvironment.

## Results

### Fabrication of the MSN+miR-146a complex

**Fig. 1:**
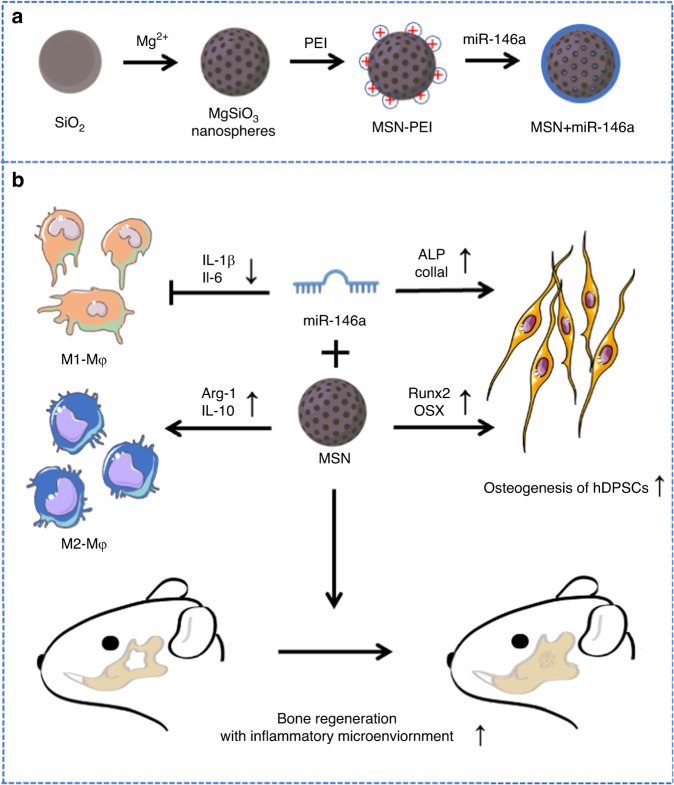
Diagram of the preparation and function of MSN+miR-146a for bone regeneration with an inflammatory microenvironment. **a** MSNs were synthesized from SiO_2_ nanoparticles and modified with PEI, and miR-146a was then loaded via physical absorption and electrostatic attraction. **b** The MSN+miR-146a complex significantly accelerated the osteogenesis of hDPSCs, reduced M1-type macrophages, increased M2-type macrophages, and promoted bone regeneration in infected mandibular bone defects in mice

**Fig. 2:**
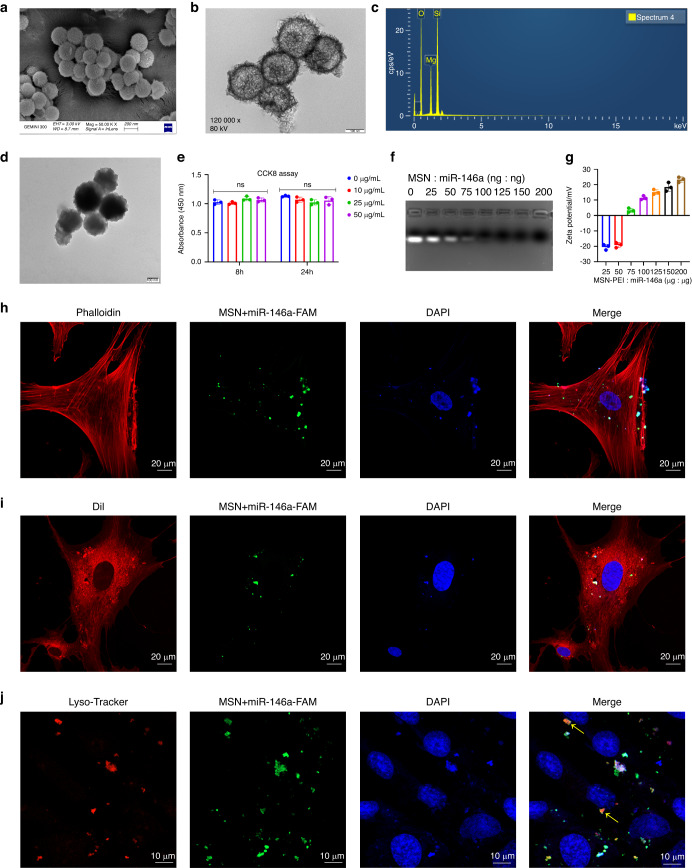
Preparation and characterization of the MSN+miR-146a complex. **a**–**c** ECM and TEM images and EDS of MSNs. **d** TEM images of MSN-PEI. **e** CCK-8 assay of hDPSCs after 8 and 24 h of coculture with PEI-modified MSNs at various concentrations. **f**, **g** Gel retardation and zeta potential tests of the MSN+miR-146a complex at different weight ratios. **h**–**j** Cellular uptake assay of MSN+miR-146a-FAM in hDPSCs after 24 h of coculture. Cells were stained to label the cytoskeleton, cell membrane or lysosome. Yellow arrows indicate colocalization of lysosomes and MSN+miR-146a-FAM. ns no significance. One-way ANOVA with Dunnett’s multiple comparisons to the blank group was used

The main results of this study are summarized in Fig. 1. MSNs were obtained after modifying classical monodispersed silica colloidal nanospheres (Fig. 1a). As shown in Fig. 2a, b, scanning electron microscopy (SEM) and transmission electron microscopy (TEM) images showed a rough surface and hollow mesoporous morphology of homogeneous MSNs with a mean diameter of 200 nm. The main elements of MSNs were determined to be magnesium, silicon and oxygen in the energy-dispersive spectroscopy (EDS) spectrum (Fig. 2c). Figure 2d shows the changed morphology of MSNs after polyethyleneimine (PEI) modification. The toxicity of MSN-PEI in hDPSCs was then validated. As shown in Fig. 2e, the viability of hDPSCs in the CCK-8 test exhibited no differences from that of the blank control group after coculture with MSNs for 48 h at a concentration of 10, 25 or 50 μg/mL, indicating good biocompatibility of MSNs even after PEI modification. Then, a gel retardation assay showed that when the amount of MSNs was increased, more miR-146a remained in the well, and only a negligible amount of miR-146a was released when the weight ratio of MSN:miR-146a was greater than 75:1 (Fig. 2f). The zeta potential of the MSN+miR-146a complex gradually switched from negative to positive when the amount of MSNs was increased (Fig. 2g). These results suggested that the optimal miR-146a-loading capacity of MSNs was at a 75:1 weight ratio. To evaluate the cellular uptake of the MSN+miR-146a complex, we mixed FAM-conjugated miR-146a with MSNs at the optimal weight ratio and then cocultured them with hDPSCs for 24 h before thorough washing with PBS. As shown in Fig. 2h, i, the MSN+miR-146a complex was endocytosed into hDPSCs with punctate green fluorescent signals in the cytoplasm indicated by cytoskeletal (phalloidin) or cell membrane (Dil probe) staining. Moreover, lysosomal staining showed colocalization of lysosomes and MSN+miR-146a-FAM, which indicated that the complex was partly encapsulated in lysosomes after internalization.

### MSN+miR-146a promoted osteogenic differentiation of hDPSCs

As pluripotent stem cells, hDPSCs can differentiate into multiple tissue cells, including osteoblasts, under classical osteogenic induction. To determine the effects of both MSNs and miR-146a on the osteogenic differentiation of hDPSCs, we used commercial liposomes, Interferin (Polyplus, France), and MSNs to transfect miR-146a separately, with nonsense miRNA chains as a negative control (NC). The transfection efficiency of miR-146a into hDPSCs was measured by quantitative reverse transcription-polymerase chain reaction (qRT‒PCR) after 7 days of osteogenic induction culture. As shown in Fig. S1a, the level of miR-146a in the miR-146a group or MSN+miR-146a group was significantly greater than that in the corresponding control group. Notably, the miR-146a level was significantly increased in the MSN group compared to the NC group, indicating an effect of MSNs on promoting endogenous miR-146a expression.

**Fig. 3:**
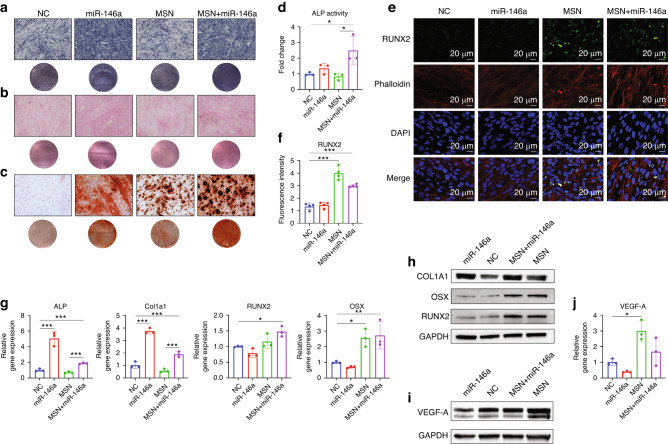
MSNs+miR-146a improved the osteogenic differentiation of hDPSCs. **a**, **b** ALP staining and SR staining of hDPSCs after 7 days of osteogenic culture. **c** ARS staining of hDPSCs after 14 days of osteogenic culture. **d** Enzyme activity assay of intracellular ALP expression in hDPSCs after 7 days of osteogenic culture. **e**, **f** IF images and quantitative analysis (*n* = 4) of RUNX2 (green) in hDPSCs after 7 days of osteogenic culture. **g**, **h** qRT‒PCR (*n* = 3) and WB results of osteogenic biomarker expression in hDPSCs after 7 days of osteogenic culture. **i**, **j** WB and qRT‒PCR (*n* = 3) results of the expression of VEGF-A in hDPSCs after 24 h of coculture with LPS-stimulated BMM-derived conditioned medium. *P < 0.05, **P < 0.01, ***P < 0.001. One-way ANOVA with Tukey’s multiple comparisons test among all groups was used

As biomarkers in osteogenesis, alkaline phosphatase (ALP) staining and Sirius red (SR) staining showed that miR-146a significantly upregulated the expression of ALP and type I collagen (Col1a1) in hDPSCs after 7 days of osteogenic induction, which was not affected by MSNs themselves (Fig. 3a, b). Then, the enzymatic activity of intracellular ALP was determined by a specific enzyme reaction, which was slightly increased in the miR-146a group but strongly increased in the MSN+miR-146a group (Fig. 3d). The western blot (WB) results also supported the upregulation effect of miR-146a on Col1a1 expression at the protein level (Fig. 3h). qRT‒PCR also confirmed the promoting effect of miR-146a on ALP and Col1a1 mRNA expression (Fig. 3g). Finally, for definite identification of osteogenic differentiation of stem cells, extracellular matrix mineralization was evaluated by typical Alizarin red S (ARS) staining. ARS distinctly increased in both the miR-146a group and MSN group and rose to the highest level in the MSN+miR-146a group after 14 days of osteogenic induction, indicating a synergic promoting effect of the two components, especially in the late phase of osteogenic differentiation of hDPSCs (Fig. 3c). On the other hand, the mRNA and protein expression of the critical osteogenic transcription factors RUNX2 and Osterix (OSX) was found to be significantly upregulated in the MSN-treated groups compared with the non-MSN groups, but no evident difference was observed between the miR-146a-treated group and the corresponding NC group (Fig. 3g, h). Higher expression of RUNX2 was also observed in the MSN-treated groups after 7 days of osteogenic induction via immunofluorescence (IF) assays (Fig. 3e, f).

Interestingly, when assessing the effect of MSN+miR-146a on the immunoregulatory function of hDPSCs, we found that MSNs significantly upregulated the expression of vascular endothelial growth factor-A (VEGF-A) when cocultured with LPS-treated BMM-derived conditioned medium for 24 h. However, this effect was potently inhibited by exogenous miR-146a, which decreased the expression of VEGF-A to a normal level, similar to that of the NC group (Fig. 3i, j and Fig. S3).

### MSN+miR-146a regulated the polarization of macrophages and osteoclast formation

In an inflammatory microenvironment, macrophages play a crucial role in bone immunity. Here, we investigated the effect of MSN+miR-146a on mouse BMM polarization under LPS stimulation with the same miRNA transfection protocol as that used for hDPSCs. Mouse BMM purity (>95%) was confirmed by both F4/80 and CD11b dual biomarkers (Fig. S2a). As shown in Fig. S1b, miR-146a expression was significantly upregulated after transfection by either liposomes or MSNs in BMMs such as hDPSCs. Nevertheless, MSNs themselves did not affect the expression of endogenous miR-146a in BMMs.

**Fig. 4:**
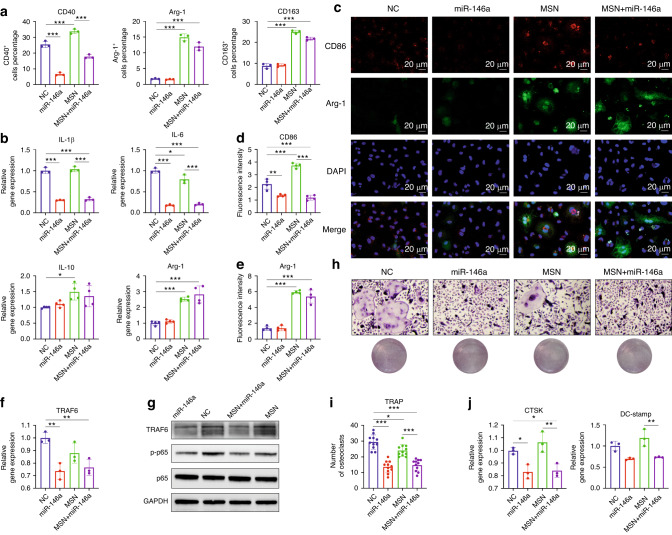
MSNs+miR-146a regulated BMM polarization and osteoclast formation. **a** Statistical results of flow cytometry of CD40-, Arg-1- or CD163-marked BMMs in the four groups after 24 h of LPS stimulation (*n* = 3). **b** The mRNA expression of IL-1β, IL-6, Arg-1 and IL-10 after 24 h of LPS stimulation (*n* = 3). **c**–**e** IF images and quantitative analysis of CD86 (red)- and Arg-1 (green)-marked BMMs after 24 h of LPS stimulation (*n* = 4). **f**, **g** qRT-PCR (*n* = 3) and WB results of the expression of TRAF6 and phosphorylation level of p65 in BMMs after 24 h of LPS stimulation. **h**, **i** TRAP staining and quantitative analysis (*n* = 11) of osteoclasts in the four groups after 6 days of RANKL induction. **j** The mRNA expression of CTSK and DC-stamp in osteoclasts after 6 days of RANKL induction (*n* = 3). *P < 0.05, **P < 0.01, ***P < 0.001. One-way ANOVA with Tukey’s multiple comparisons test among all groups was used

As shown in Fig. 4b and Fig. S2b, after 24 h of 1 μg/mL LPS stimulation, the proportion of CD40^high^ M1-polarized BMMs decreased significantly in the miR-146a group and the MSN+miR-146a group compared to the corresponding control group. On the other hand, the proportion of arginase 1 (Arg-1)^high^ or CD163^high^ M2-polarized BMMs apparently rose to higher levels in the MSN-treated groups than in the non-MSN groups. Although the proportion of CD40^high^ M1 BMMs increased with only MSNs, this effect could be reversed by loading miR-146a, resulting in a lower level than that in the NC group. Similar trends in BMM polarization changes were further confirmed by IF staining with CD86 as an M1 marker and Arg-1 as an M2 marker (Fig. 4c–e). Accordingly, the mRNA expression of the critical proinflammatory cytokines interleukin (IL)-1β and IL-6 was potently downregulated by miR-146a, while the mRNA expression of Arg-1 increased significantly in the MSN-treated groups compared with the non-MSN groups, with a similar but weaker trend detected for IL-10 expression (Fig. 4b). The effect of MSN+miR-146a on the activation of the canonical NF-κB pathway was examined. The expression of TRAK6 at both the mRNA and protein levels was significantly downregulated by miR-146a, and the phosphorylation level of p65 decreased as well, indicating that the NF-κB pathway was inhibited by miR-146a in mouse macrophages (Fig. 4f, g).

In addition, the effect of MSN+miR-146a on osteoclast formation was evaluated. In vitro tartrate-resistant acid phosphatase (TRAP) staining experiments showed that miR-146a strongly inhibited osteoclast differentiation and maturation of osteoclast progenitor cells from mouse bone marrow (Fig. 4h, i), and the mRNA expression of cathepsin K (CTSK) and dendritic cell-specific transmembrane protein (DC-stamp) was significantly downregulated by miR-146a (Fig. 4j).

### MSN+miR-146a accelerated bone regeneration in simulated infected mouse mandibular defects

**Fig. 5:**
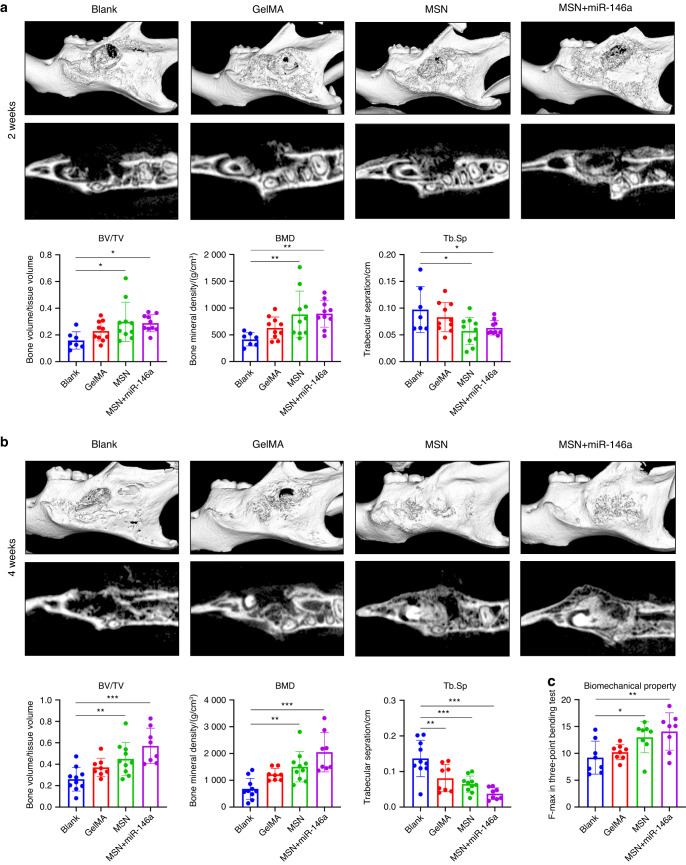
MSNs+miR-146a accelerated bone regeneration in a stimulated infected mouse mandibular defect model. **a**, **b** Micro-CT examinations of mouse mandible samples after 2 weeks (**a**) and 4 weeks (**b**) of healing following surgery with representative reconstructed volume and slice images and the mean BV/TV, BMD and Tb.Sp (*n* = 7–10). **c** Maximal force detected in the three-point bending test of trimmed mouse mandible samples (*n* = 8). *P < 0.05, **P < 0.01, ***P < 0.001. One-way ANOVA with Dunnett’s multiple comparisons to the blank group was used

To investigate the effect of the MSN+miR-146a complex on in vivo osteogenesis, we generated mouse-infected mandibular bone defect models, and the biomaterials were delivered by a commercial photocuring hydrogel, GelMA. As shown in Fig. 5a, after 2 weeks of uneventful healing, an oval defect could still be observed in all mandibles, with more calcified new bone detected in the MSN group and MSN+miR-146a group. Quantitative analysis showed that compared to those of the blank group, both the bone volume/total volume (BV/TV) and bone mineral density (BMD) of new bone increased slightly in the GelMA group but increased substantially in the MSN group and MSN+miR-146a group, while separation of trabecular bone (Th. Sp) showed the opposite trend. After 4 weeks of healing, the MSN group and MSN+miR-146a group showed better healing with more regenerated high-density mineralized tissue in the defect area. Similarly, the BV/TV and the BMD increased in the three experimental groups but most significantly in the MSN+miR-146a group, with Tb.Sp revealing the opposite trend (Fig. 5b). The biomechanical properties of the bone defect region after 4 weeks of healing were tested via a three-point bending test, which showed that the mean maximal force detected in the bending process was significantly increased in the MSN group and MSN+miR-146a group (Fig. 5c).

**Fig. 6:**
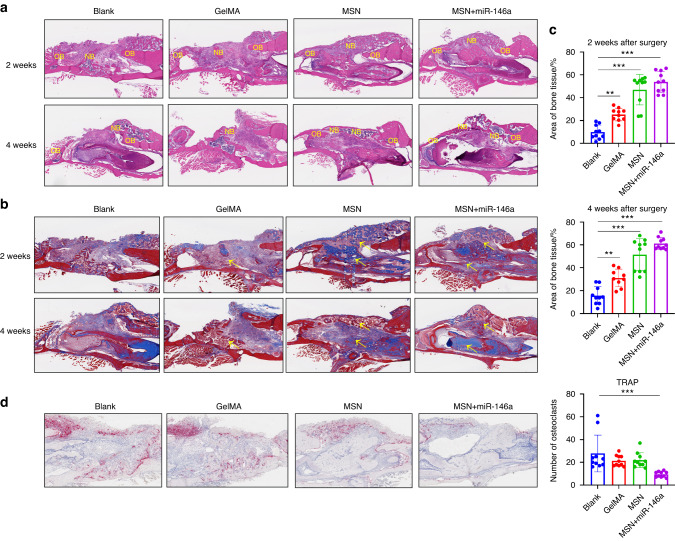
MSNs+miR-146a promoted bone regeneration but inhibited osteoclast formation. **a** HE staining of the mouse mandible samples after 2 and 4 weeks of healing. NB new bone, OB original bone. **b** Masson staining of the samples after 2 and 4 weeks of healing. Yellow arrows indicate blue-stained type I collagen of new bone. **c** Statistical analysis of the area proportion of bone tissues in Masson-stained slices of the samples after 2 and 4 weeks of healing (*n* = 9–10). **d** TRAP staining and quantitative analysis (*n* = 10) of the samples after 2 weeks of healing. *P < 0.05, **P < 0.01, ***P < 0.001 One-way ANOVA with Dunnett’s multiple comparisons to the blank group was used

Further histological results also supported the osteogenic-promoting effect of the MSN+miR-146a complex. As shown in Fig. 6a, much more premature bone tissue was observed in the defect area in the MSN group and MSN+miR-146a group after 2 weeks of healing than in the blank group and GelMA group, which was more distinct with blue-stained collagen in the Masson staining results (Fig. 6b). Similarly, the MSN group and MSN+miR-146a group recovered better with relative contact with the outer bone plate 4 weeks after surgery, as revealed by the HE and Masson staining results (Fig. 6a, b). The semiquantitative analysis of the bone tissue area supported the beneficial effects of MSNs and miR-146a on bone regeneration (Fig. 6c), with the highest proportion of hard tissue detected in the MSN+miR-146a group. In vivo osteoclast formation was assessed simultaneously in the 2 week healing groups. Only in the MSN+miR-146a group did the formation of TRAP+ osteoclasts significantly decrease in the simulated infected bone defects (Fig. 6d).

**Fig. 7:**
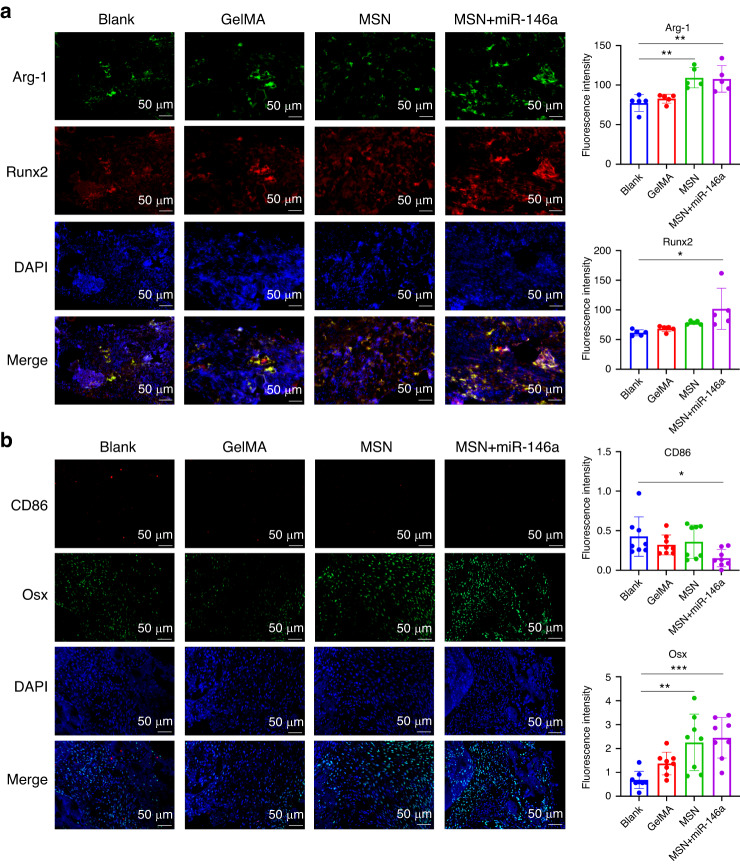
MSNs+miR-146a regulated the in vivo polarization of macrophages and promoted osteogenic marker expression. **a** IF images and quantitative analysis (*n* = 5) of the expression of Arg-1 (green) and Runx2 (red) in the defect area of mouse mandible samples after 2 weeks of healing. **b** IF images and quantitative analysis (*n* = 8) of the expression of CD86 (red) and Arg-1 (green) in the defect area of the samples after 2 weeks of healing. *P < 0.05, **P < 0.01, ***P < 0.001. One-way ANOVA with Dunnett’s multiple comparisons to the blank group was used

IF staining revealed the immunoregulatory and osteogenic-promoting effects of the MSN+miR-146a complex. As shown in Fig. 7a, after 2 weeks of healing, a lower level of CD86^high^ M1 BMMs was observed in the MSN+miR-146a group, but a significantly higher level of Arg-1^high^ M2 BMMs was found in the MSN group and the MSN+miR-146a group. The expression of Runx2 was significantly increased in the MSN+miR-146a group, and the expression of OSX was evidently increased in both the MSN group and MSN+miR-146a group (Fig. 7b). These results cumulatively suggested that with the help of the GelMA hydrogel, MSN+miR-146a could promote early osteogenesis in stimulated infected mouse mandibular defects with immunoregulatory capacity in macrophages.

## Discussion

In this study, we comprehensively investigated the application potential of the MSN+miR-146a complex in treating irregular bone defects with an inflammatory microenvironment. The biocompatibility and endocytosis ability of pure MSNs have been confirmed in our previous studies.^16–18^ Here, PEI, a polymer with a high density of positively charged amino groups, was utilized to increase the oligo-loading capacity of MSNs.^21^ PEI-modified MSNs remained favorable for biocompatibility, and the optimal weight ratio of MSNs and miR-146a was determined to be 75:1, giving the complex a positive surface charge to better bind with the negatively charged cell membrane.^22^

HDPSCs, first isolated in 2000,^23^ appear to be a promising stem cell resource with high proliferation, multidifferentiation potential,^24,25^ and easier surgical access with less risk of donor site morbidity.^26,27^ In this study, miR-146a and MSNs were observed to accelerate the in vitro osteogenesis of hDPSCs in different ways. By directly downregulating the tumor necrosis factor α-induced NF-κB pathway, miR-146a was reported to block its negative influence on the osteogenic differentiation of M3CT3-E1 preosteoblasts,^20^ indicating that the promoting effect of miR-146a on osteogenesis may be associated with the classical NF-κB pathway. However, the expression of ALP and Col1a1 was not affected by MSNs and appeared to be lower in the MSN+miR-146a group than in the miR-146a group. This result might be attributed to the slower intracellular release of miRNA from high-absorbability hollow nanospheres than from commercial liposomes. Di et al. (2018) reported that by directly targeting Dickkopf-related protein 1, the negative feedback inhibitor in Wnt/β-actin signaling, miR-146a knockout could reverse the excess osteogenic potential in ankylosing spondylitis fibroblasts.^13^ However, the speculated upregulation of RUNX2 via the Wnt/β-actin pathway after miR-146a overexpression was not observed in hDPSCs, which needs further investigation.

After being internalized, MSNs can be degraded by lysosomes and sustainably release silicon and magnesium ions. Although the silicon-based biomaterial was reported to upregulate endogenous expression of miR-146a in hBMSCs,^20^ this effect was weak when compared to the magnitude of exogenous miR-146a. On the other hand, magnesium ions were reported to improve the expression of RUNX2 by activating the Wnt/β-actin pathway.^19^ OSX, a zinc finger transcription factor essential to osteogenesis that acts downstream of RUNX2,^28^ showed similar significantly upregulated expression in MSN-treated groups, as expected. Interestingly, more mineralized nodules were observed in the MSN-treated groups, suggesting that MSNs might also act as self-standing cores for matrix mineralization. Higher in vivo expression of Runx2 and Osx was also observed in stimulated infected mouse mandibular defects after administration of MSNs, although the upregulation of Runx2 only showed a significant difference in the MSN+miR-146a group. Potent immunoregulatory functions indicate that hDPSCs have more application potential in treating inflammatory bone defects.^29^ Surprisingly, MSNs upregulated the expression of VEGF-A in hDPSCs in a simulative inflammatory microenvironment. However, the effect was strongly eliminated by miR-146a, implying a potential shortage of this nucleic acid drug when considering angiogenesis in bone regeneration.^30^ In further studies, appropriate molecular drugs may be combined with MSNs to enhance the indirect proangiogenic effect of MSNs.

Macrophages play a crucial role in maintaining homeostasis, including bone tissues.^31^ As an anti-inflammatory miRNA,^11^ miR-146a was reported to be endotoxin-responsive and targeted critical inflammation-related proteins in human monocytes, including TRAF6, IRAK1 and TLR4.^12,32^ As expected, both in vitro and in vivo M1-polarized BMMs significantly decreased after treatment with miR-146a, which could be attributed to the negative post-translational repression of miR-146a on TRAF6, a crucial intracellular factor that mediates activation of the NF-κB pathway when TLR4 binds specific antigens such as LPS.^33^ The lower phosphorylation level of p65 supported the negative regulation of miR-146a on the canonical NF-κB pathway in mouse macrophages. Notably, M1-type BMMs were relatively increased due to MSN addition, which might be a result of foreign body reactions elicited in macrophages by nanoparticle biomaterials,^34^ but the inevitable side effect could be finally decreased by miR-146a to a lower level. Interestingly, MSNs significantly increased the proportion of both in vitro and in vivo M2-type BMMs even under high-concentration LPS conditions. It is widely believed that an in-time shift of the macrophage population from a proinflammatory to a proregenerative phenotype is beneficial to bone regeneration after injury,^33,35^ and the immunomodulatory effect of MSNs gives biomaterials additional potential in treating bone defects with chronic inflammation.

High levels of proinflammatory cytokines such as TNF-α and sustained activation of NF-kB signaling are thought to be critical reasons for impaired bone regeneration in chronic inflammatory conditions,^36^ which could result in hyperactive osteoclast formation as well. Here, miR-146a potently inhibited in vitro and in vivo osteoclast formation, which may be attributed to the same downregulating effect on TRAF6.^37,38^ This result suggested the therapeutic potential of MSNs in bone regeneration with an inflammatory microenvironment through regulation of the osteogenesis-osteoclasis balance.

There are some limitations of this study. Although the application value of hDPSCs has been widely investigated in bone engineering, the promoting effect of MSNs+miR-146a on the in vitro osteogenesis of hDPSCs should be carefully interpreted, and further studies that incorporate stem cells into the biomaterial system are needed. In addition, the LPS-stimulated mouse-infected mandibular defect model could not fully simulate clinical cases of oral-maxillofacial bone defects resulting from microbial infection, such as periodontitis. Therefore, large animal bone defect models with pre-existing periodontitis or a severe bacterial infection should be built. Due to the hollow mesoporous structure and high adsorption capacity, MSNs could load multiple molecular drugs together with miR-146a to create multifunctional nanomaterial systems, which included benidipine (an antihypertensive drug) that was demonstrated to promote osteogenesis and angiogenesis.^18,39^

## Conclusion

By loading the multifunctional nucleic acid drug miR-146a, the pro-osteogenic nanomaterial MSNs successfully accelerated the osteogenic differentiation of hDPSCs and new bone formation in stimulated infected mouse mandibular bone defects with satisfying immunoregulatory capacity in macrophage polarization. The MSN+miR-146a biomaterial complex delivered by the photocuring hydrogel showed good therapeutic potential in treating irregular bone regeneration with an inflammatory microenvironment.

## Materials and methods

### Materials and reagents

Ammonium hydroxide aqueous solution (NH_3_·H_2_O, 28%), ethanol, tetraethyl orthosilicate (TEOS), magnesium chloride hexahydrate (MgCl_2_·6H_2_O) and ammonia chloride (NH_4_Cl) were all purchased from Aladdin Biochemical Technology Co., Ltd. (China), and polyethyleneimine (PEI, mw = 25 000) was purchased from Sigma-Aldrich (USA).

### Preparation and characterization of MSNs

Magnesium silicate nanospheres (MSNs) were synthesized via a two-step route in accordance with our previous studies.^17^ Monodispersed silica colloidal nanospheres (nano-SiO_2_) with an average diameter of 200 nmol/L were prepared with the modified Stöber method. MgCl_2_·6H_2_O (304.50 mg), NH_4_Cl (1.07 g) and NH_3_·H_2_O (2.00 mL) were dissolved in 20 mL of deionized water. Nano-SiO_2_ (200 mg) was dispersed in another 20 mL of deionized water with ultrasonic oscillation. Then, the two solutions were mixed and transferred into a reaction still and sealed to heat at 160 °C for 12 h. After naturally cooling to room temperature, the obtained MSNs were rinsed with deionized water and ethanol in turn and dried in vacuum at 60 °C overnight. The morphological and structural features of MSNs were examined by SEM (GeminiSEM 300, Zeiss, Germany) and TEM (JEM-1400flash, JEOL, Japan), and EDS was used to detect the main elements in MSNs.

### Modification of MSNs and preparation of the MSN+miR-146a complex

MSNs were modified with PEI via electrostatic interactions.^21^ MSNs were resuspended in deionized water (1 mg/mL) and added to an equivalent PEI solution (1 mg/mL), and the mixed solution was stirred at room temperature at 400 r/min for 3 h. After centrifugation and thorough washing with deionized water, MSN-PEI was prepared and stored in deionized water at a 1 mg/mL concentration and examined by TEM. MiR-146a in this study is referred to as miR-146a-5p, and its sequence is shown in Table S1. MSN solutions (10 μL) were combined with miR-146a solutions (0.05 μg, 10 μL) at various weight ratios (MSN:miR-146a = 0:1, 25:1, 50:1, 75:1, 100:1, 125:1, 150:1, 200:1), mixed with a vortex mixer for 1 min and then incubated at 4 °C for 30 min. The optimal loading ratio was confirmed using a gel retardation assay with 1% agarose gel containing 1 × GelRed (Us Everbright, USA). The MSN+miR-146a complex was added to 10 × loading buffer, and electrophoresis was carried out at 100 V for 10 min in TAE running buffer (Mei5Bio, China). The miR-146a gel was visualized with a ChemiDoc MP Imaging System (Bio-Rad, USA). Then, the surface charge of MSNs adsorbing various amounts of miR-146a was estimated by a zeta potential analysis meter (Surpass, Anton Paar, Austria).

### Cell culture

hDPSCs were isolated from third molars (clinical waste) or permanent teeth from adolescents following reported protocols,^40^ which were approved by the Ethical Committee of Stomatology Hospital of Zhejiang University School of Medicine (ethics approval number: 2023-027) in accordance with the Helsinki Declaration. Written informed consent was obtained from the subject or subject’s parents. Briefly, dental pulp was extracted with a dentinal excavator and then gently rinsed with phosphate-buffered solution (PBS) (Cienry, China). After being dissected into 1–2 mm^3^ pieces, the dental pulp tissue was planted into a 25 mm^2^ culture flask containing 1 mL of fetal bovine serum (FBS) (Gibco, USA) and cultured in a humidified incubator at 37 °C with 5% CO_2_ for 6 h. Then, 0.5 mL of minimum essential medium α (MEM α) containing 2 mmol/L L-glutamine (Gibco, USA) with 10% FBS and 100 U/mL streptomycin/penicillin (Cienry) (complete MEM α) was added into the flask for further culture. The medium was gently changed every 3 days, and the cells were passaged until 80% confluence using 0.05% trypsin containing ethylenediaminetetraacetic acid (EDTA) (Thermo Fisher, USA). hDPSCs after P3 were collected for the study.

Mouse BMMs were harvested from the femur and tibia bones of 5-week-old male C57BL/6J mice (Zhang et al., 2008). The bone marrow was flushed out using a 25G needle and 1 mL syringe filled with cold MEM α until the bones turned pale. The turbid cell liquid was pipetted up and down and then filtered through a 70 μm filter. After centrifugation at 250 g for 5 min, the supernatant was discarded, and the cell pellet was resuspended and incubated in ammonium-chloride-potassium (ACK) lysing buffer (Amizona, USA) for 90 s to remove red blood cells. After PBS washes and centrifugation, the cells were resuspended in complete MEM α with 20 ng/mL recombinant mouse macrophage colony-stimulating factor (M-CSF) (Amizona) (MΦ-MEM α). The cells were counted and directly seeded at 1 × 10^5^ cells/well into 24-well plates. The same BMMs were treated with 40 ng/mL M-CSF and an additional 40 ng/mL recombinant mouse receptor activator of nuclear factor kappa-B ligand (RANKL) (Amizona) to induce osteoclast differentiation. All animal procedures (including following in vivo experiments) were approved by the Institutional Animal Care and Use Committee of Zhejiang Center of Laboratory Animals (approval No. ZJCLA-IACUC-20010204).

### Cytotoxic assay of MSNs

hDPSCs were seeded at 2 × 10^3^ cells/well in 96-well plates. The next day, MSNs were added at various final concentrations (0–50 μg/mL). After coculture for 8 and 24 h, the CCK-8 assay was performed using a CCK-8 Cell Proliferation Kit (Beyotime, China) in accordance with the manufacturer’s instructions.

### Cellular uptake assay

hDPSCs were seeded at 1.5 × 10^3^ cells/well on glass coverslips in 24-well plates. The next day, 25 μg/mL MSN+miR-146a-FAM complex (MSN:miR-146a-FAM = 75:1) was added and cocultured with hDPSCs for 24 h. Then, the cells were fixed with 4% (v/v) paraformaldehyde (Haoke Biotech, China) and permeabilized with 0.1% (v/v) Triton X-100 (Servicebio, China). The cells were stained with rhodamine-labeled phalloidin (Invitrogen, USA) to label the cytoskeleton, a DiI probe (Beyotime) to label the cell membrane or LysoTracker Red (Beyotime) to label lysosomes before mounting with DAPI (Servicebio). Samples were imaged using a laser scanning confocal microscope (LSM980, Zeiss).

### Transfection of miR-146a and in vitro cell experiments

All cells were divided into four groups: the miR-146a group and NC group (purely transfected with miRNA by lipidosome), MSN group (loading nonsense oligo) and MSN+miR-146a group. hDPSCs were seeded at 1.5 × 10^4^ cells/well in 24-well plates. When cells became confluent, in the non-MSN groups, 20 nmol/L miR-146a or NC miRNA was transfected with Interferin, while the corresponding MSN+miR-146a or MSN-NC complex (MSN: miRNA = 75:1) was added to the MSN-treated groups. After 6 h of culture, all hDPSCs were thoroughly washed in PBS and incubated in fresh complete MEM α with 50 μg/mL ascorbic acid (Mecklin, China), 10 mmol/L *β*-glycerophosphate (Sangon Biotech, China) and 100 nmol/L dexamethasone (Mecklin) (osteogenic MEM α). The medium was changed every 3 days. The osteogenic differentiation of hDPSCs was evaluated by specific in vitro staining using an ALP staining kit (Beyotime, China), ALP activity assay kit (Beyotime) and SR staining kit (Phygene, China) on Day 7 followed by an ARS staining kit (Beyotime) on Day 14 according to the manufacturer’s instructions.

For BMMs, the medium was first changed 2 days after plating, and miR-146a and NC miRNA were transfected with Interferin or MSNs similarly for 6 h. Then, fresh MΦ-MEM α containing 1 μg/mL LPS (Mei5bio) was added to induce the polarization of BMMs. The supernatant was collected and directly added to another culture plate of hDPSCs (already received the same miRNA transfection as above) for 24 h to stimulate an inflammatory microenvironment for hDPSCs. For osteoclasts, the transfection of miRNA was performed on day 3 after M-CSF and RANKL treatment, and the medium was first changed on day 4. Osteoclastic differentiation was evaluated by a TRAP staining kit (Amizona).

### Quantitative RT‒PCR

hDPSCs were collected after culture in osteogenic MEM α for 7 days or coculture with LPS-stimulated BMM-derived conditioned medium for 24 h, and BMMs were collected after 24 h of 1 μg/mL LPS stimulation. Total RNA was extracted with an RNA extraction kit (Vazyme, China) and quantified with a Nanodrop 3000 (Thermo Fisher). Reverse transcription (RT) of mRNA was performed with 400 ng RNA using a cDNA synthesis kit (Vazyme), and RT of miRNA was performed with 100 ng RNA using a miRNA synthesis kit (Accurate Biology, China). Then, qPCR was carried out on QuantStudio 7 Flex (Life Technology, USA) using HiScript II Q RT SuperMix for qPCR (Vazyme) in a 10 μL reaction volume. β-actin and U6 were used as the endogenous reference genes. The sequences of the primer pairs are presented in Table S1. The relative gene expression level of target genes was calculated using the ΔΔCt method.

### Western blotting

HDPSCs after 7 days of osteogenic induction and BMMs after 24 h of 1 μg/mL LPS stimulation were lysed in RIPA lysis buffer (Beyotime) for 30 min on ice, ultrasonicated for 3 s, and then centrifuged at 15 000 × *g* at 4 °C for 5 min. The protein concentration of the supernatant was determined using a BCA protein kit (Beyotime). After the samples was mixed with 5 × SDS loading buffer (Mei5bio) to obtain 1 μg/μL protein solution, sodium dodecyl sulfate–polyacrylamide gel electrophoresis was conducted to separate proteins with 10 μg per lane. Then, the protein was transferred to a 0.2 μm polyvinylidene difluoride (PVDF) membrane (Sigma-Aldrich). After being blocked in 5% defatted milk for 50 min at room temperature, the membrane was incubated at 4 °C overnight with primary antibodies against the following proteins: β-actin (1:10 000, 66009-1-Ig) and GAPDH (1:10 000, 60004-1-Ig) (both from Proteintech, USA); Col1a1 (1:1 000, 720260S), TRAF6 (1:500, 67591), p65 (1:500, 8242) and p-p65 (1:500, 3033) (all from Cell Signaling, USA); OSX (1:1 000, ab209484) and VEGF-A (1:1 000, ab214424 (both from Abcam, USA); and RUNX2 (1:500, ET1612-47, Huabio, China). Following a 1 h incubation with secondary horseradish peroxidase (HRP)-conjugated anti-mouse or anti-rabbit IgG antibodies (1:10 000, SA00001-1 and SA00001-2, Proteintech) at room temperature, the membrane was visualized with an enhanced luminol-based chemiluminescent (ECL) kit (Thermo Fisher) and exposed in a ChemiDoc MP Imaging System (Bio-Rad).

### Flow cytometry

BMMs were collected after 24 h of 1 μg/mL LPS stimulation using 0.05% trypsin and resuspended in PBS after centrifugation. Flow cytometry staining was performed according to the manufacturer’s instructions. PE F4/80 antibody (123109) and PerCP/Cy5.5 CD11b antibody (101227) (both from BioLegend, USA) were used to mark mouse macrophages. BV421 CD40 antibody (562846, BD Pharmingen, USA), Alexa Fluor (AF) 488 Arg-1 antibody (53-3697-82) and SB436 CD163 antibody (62-1631-82) (both from Thermo Fisher) were used to stain BMMs as markers of M1 or M2 polarization for 30 min on ice. Data acquisition was performed using CytoFlex (Beckman Coulter, USA) and analyzed with CytoExpert software (Beckman Coulter).

### Cell immunofluorescence assay

HDPSCs after 7 days of osteogenic induction and BMMs after 24 h of 1 μg/mL LPS stimulation were washed thoroughly with PBS, fixed in 4% (v/v) paraformaldehyde and permeabilized with 0.1% (v/v) Triton X-100. The hDPSCs were stained with RUNX2 antibody (1:5 000, 1256S, Cell Signaling) overnight at 4 °C and incubated with secondary AF488 anti-rabbit antibody (1:500, 21441, Thermo Fisher) for 2 h at room temperature. BMMs were stained with CD86 (1:500, 26903-1-AP, Proteintech) or AF488 Arg-1 antibody (1:500, 53-3697-82, Thermo Fisher) overnight at 4 °C followed by secondary CoraLite 594 anti-rabbit antibody (for CD86) (1:500, SA00013-4, Proteintech) for 2 h at room temperature. The cytoskeleton was stained with rhodamine-labeled phalloidin if needed before mounting with DAPI. Samples were imaged using an LSM980 laser scanning confocal microscope, and the mean IF intensity was quantitatively measured by ImageJ software.

### Mouse mandibular bone defect model

Eight-week-old male C57BL/6 mice were used to create mandibular bone defect models and divided into four groups: Blank, GelMA, MSN, MSN+miR-146a (*n* = 10 each). GelMA refers to a commercial gelatin methacryloyl hydrogel (EFL-GM 90, Engineering for Life, China). A 5% GelMA solution containing 1 mg/mL MSN with or without a corresponding amount of miR-146a was freshly prepared. Mice were anesthetized by intraperitoneal injection of 2,2,2-tribromoethanol (200 mg/kg; Sigma-Aldrich, USA), and the left mandibular region was shaved and disinfected. A 5-mm-long incision was made to expose the mandibular bone, and a 2 × 1.5 × 1 mm^3^ critical-size bone defect was created using a high-speed round bur. Approximately 5 μL of gel solution containing 50 μg/mL LPS and the appropriate biomaterial was injected per site and photocured with 405 nmol/L light, while the blank group was administered the same amount of PBS with LPS at the same time. The incision was carefully closed with a 5-0 silk suture. All mice were monitored every day, and no adverse effects were observed. After 2 and 4 weeks of healing, animals were euthanized using carbon dioxide, and mandible samples were collected and fixed in 4% (v/v) paraformaldehyde for 24 h followed by 70% ethanol at 4  °C.

### Microcomputed tomography (CT) assay and biomechanical test

Mouse mandible samples were scanned using micro-CT (U-CT-XUHR, Milabs, The Netherlands) at a voltage of 55 kV and current of 0.17 mA with a 75 ms exposure time. After 3D volume rendering of scan data in Imalytics software (Gremse-IT, Germany), a virtual cylinder was created (1.2 mm diameter and 1 mm height) inside the mandibular bone defect area and designated as the region of interest (ROI). The bone grayscale threshold was set at 1 400 Hounsfield units (HU) for all samples. The BV/TV, BMD and Th. Sp were calculated to compare new bone formation among groups.

Another set of mice (*n* = 8) was euthanized after 4 weeks of healing. The paraformaldehyde-fixed mandibles were trimmed into 10 × 4 mm strip-like samples and subjected to a three-point bending test performed in an electronic universal material testing machine (5943, Instron, USA). The middle probe of the machine was directly pressed on the bone defect area, and the mean maximal force detected in the bending process was recorded for statistical analysis.

### Histological and immunofluorescence analyses

The mandible samples were decalcified in a fast decalcified solution (Biotech, China) for 24 h at room temperature, dehydrated in graded ethanol and then embedded in wax. The tissue was cut into 5 μm sections using a microtome (Leica) and stained with HE, Masson’s trichrome and TRAP with commercial staining kits (Servicebio) to assess new bone formation and osteoclast function. After being blocked with bovine serum albumin (Servicebio), the sections were incubated with primary antibodies against Arg-1 (1:500, GB11285, Servicebio) and Runx2 (1:500, GB11264, Servicebio) as well as CD86 (1:500, 26903-1-AP, Proteintech) and Osx (1:500, ab209484, Abcam) at 4 °C overnight. Then, the sections were incubated with appropriate secondary antibodies (Servicebio) before mounting with DAPI. Samples were imaged using a DMI8 inverted fluorescence microscope (Leica Microsystems, Germany) and an LSM980 laser scanning confocal microscope with quantitative analysis by ImageJ.

### Data analysis

Data were analyzed using Prism 8.0 software (GraphPad, USA) and are represented as the mean ± standard deviation (SD). Student’s *t*-test or one-way analysis of variance (ANOVA) was used to analyze the differences among groups followed by Tukey’s or Dunnett’s multiple comparison tests. The statistical significance level was set at *P* < 0.05.

### Supplementary information

Supplementary information
