# Supplementary material for: MicroRNA-146a-loaded magnesium silicate nanospheres promote bone regeneration in an inflammatory microenvironment
Source: Bone Res. 2024 Jan 15;12:2. doi: 10.1038/s41413-023-00299-0 (PMC10788347; doi:10.1038/s41413-023-00299-0)
Supplement: Supplementary file 1 — Supplementary information [file 41413_2023_299_MOESM1_ESM.docx]

|  | Forward sequence | Reversed sequence |
| --- | --- | --- |
| miR146a-5p | CTGAGAACTGAATTCCATGGGTT |  |
| Human |  |  |
| β-actin | CATGTACGTTGCTATCCAGGC | CTCCTTAATGTCACGCACGAT |
| U6 | CTCGCTTCGGCAGCACA | AACGCTTCACGAATTTGCGT |
| ALP | CCAACTCTTTTGTGCCAGAGA | GGCTACATTGGTGTTGAGCTTTT |
| Col1a1 | GAGGGCCAAGACGAAGACATC | CAGATCACGTCATCGCACAAC |
| OSX | CAGCAGCTAAACTTGGAAGGA | TGCTTTCGCTTGTCTGAGTC |
| RUNX2 | TGGTTACTGTCATGGCGGGTA | TCTCAGATCGTTGAACCTTGCTA |
| VEGF-A | AGGGCAGAATCATCACGAAGT | AGGGTCTCGATTGGATGGCA |
| Mouse |  |  |
| β-actin | GGCTGTATTCCCCTCCATCG | CCAGTTGGTAACAATGCCATGT |
| U6 | CTCGCTTCGGCAGCACA | AACGCTTCACGAATTTGCGT |
| IL-1β | GAAATGCCACCTTTTGACAGTG | TGGATGCTCTCATCAGGACAG |
| IL-6 | TAGTCCTTCCTACCCCAATTTCC | TTGGTCCTTAGCCACTCCTTC |
| Arg-1 | CTCCAAGCCAAAGTCCTTAGAG | AGGAGCTGTCATTAGGGACATC |
| IL-10 | GCTCTTACTGACTGGCATGAG | CGCAGCTCTAGGAGCATGTG |
| TRAF6 | AAAGCGAGAGATTCTTTCCCTG | ACTGGGGACAATTCACTAGAGC |
| CTSK | TGGGCCAGGATGAAAGTTG | CCCCACAGGAATCTCTCTGT |
| DC-stamp | GGGGACTTATGTGTTTCCACG | ACAAAGCAACAGACTCCCAAAT |

Table S1. Sequences of miR146a-5p and qPCR primers


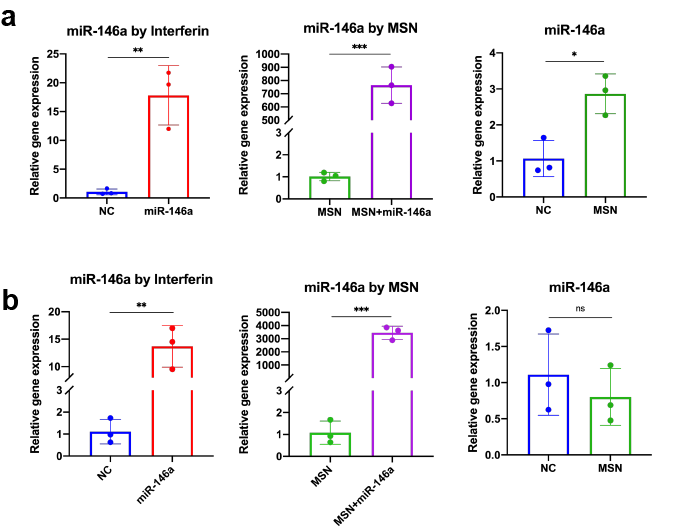


**Fig. S1** *In vitro* miR-146a transfection efficiancy of interferin and MSN. The levels of miR146a after transfection by Interferin or MSN as well as the endogenous miR146a expression in hDPSCs after 7 d osteogenic culture (**a**) and in BMMs after 24 h LPS stimulation (**b**). * P<0.05, ** P<0.01, *** P<0.001. Student t-test was used.


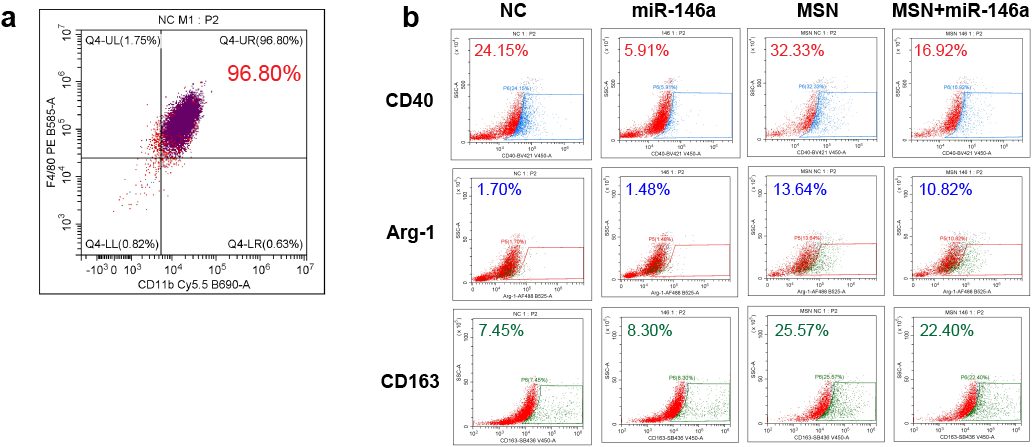


**Fig. S2** Purity and *in vitro* polarization of mouse BMMs. Flow cytometry results of F4/80^high^ and CD11b^high^ mouse macrophages in BMMs (**a**) and CD40^high^ M1-type macrophages and Arg-1^high^ or CD163^high^ M2-type macrophages in the four groups after 24 h LPS stimulation (**b**).


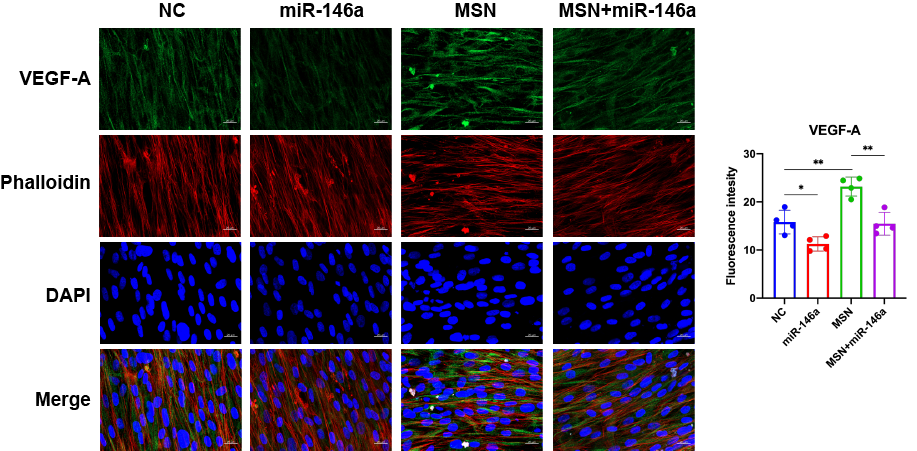


**Fig. S3** IF assay of *in vitro* VEGF-A expression in hDPSCs with inflammatory microenvironment. IF images and quantitative analysis of VEGF-A (green) expression of hDPSCs after 24 h coculture with LPS-stimulated BMMs-derived conditioned medium. * P<0.05, ** P<0.01. One-way ANOVA test with Tukey's multiple comparisons among all groups were used.
